# Editorial: Diving deeper with metabolomics into animal physiology

**DOI:** 10.3389/fmolb.2024.1503412

**Published:** 2024-11-29

**Authors:** Dagnachew Hailemariam, Elda Dervishi

**Affiliations:** ^1^ Department of Agricultural, Food and Nutritional Science, University of Alberta, Edmonton, AB, Canada; ^2^ Department of Animal and Veterinary Sciences, California State Polytechnic University Pomona, Pomona, CA, United States

**Keywords:** animal physiology, metabolomics, health, efficiency, biomarkers, genetics

This research topic brings together metabolomics based research in different animal species that include dairy cows, goats, pigs, bats, silkworms and parasitic nematodes. The studies in dairy cows, goats and pigs employed metabolomics to understand the physiology of different aspects of the animal that could be applicable to improve sustainability, animal health and welfare, antimicrobial use and fertility. Furthermore, metabolomics was used to compare the metabolites in the cochleae of two bat species. The effect of diet on cocoon yield in silkworms and developmental stage specific mechanisms of survival in parasitic nematode has been investigated using metabolomics.

Feed efficiency and reproductive management are two important aspects of dairy farming which has significant economic contribution to milk production. Hailemariam et al. investigated the physiological underpinnings of residual feed intake (a measure of feed efficiency), identified biomarkers and developed prediction models using targeted milk metabolomics. The authors reported varying mechanisms of feed efficiency during the lactation period and lactation stage-dependent biomarkers and prediction models that can assist in generating large-scale records that can be used to select dairy cows for improved feed efficiency. Some of the lactation stage specific biomarkers include decanoylcarnitine (AUC = 0.81), dodecenoylcarnitine (AUC = 0.81) and phenylalanine (AUC = 0.85) at early, mid and late stages of lactation, respectively. In another study, Pollock et al. explored the relationship between higher estrus associated temperatures (HEAT) and pre-ovulatory follicular fluid metabolome. The study revealed association of follicular fluid metabolome with HEAT, for instance, the maximum vaginal temperature was related to the differential abundances of uracil, uric acid, and 6-phospho-D-gluconate when expressed as change from the baseline. The findings support the concept that HEAT is related to changes in the pre-ovulatory follicular fluid metabolites involved in energy metabolism, thermoregulation, and oxidative stress management. The study by Vasco et al. combines the use of untargeted metabolomics and metagenomics to investigate the effect of intramammary ceftiofur treatment and different lactation stages on the metabolic and microbial profiles of dairy cattle hindgut. The authors reported that the week after treatment, treated cows had elevated levels of stachyose, phosphatidylethanolamine diacylglycerol and inosine. This research provides information into how antibiotic treatments and lactation stages influence the gut microbiome and metabolome in cattle. Furthermore, the dynamics of fecal microbiome and blood metabolites of dairy calves during the first 2 weeks after birth was investigated by Kojima et al. Early microbiomes were dominated by Proteobacteria, while Firmicutes and Bacteroidetes became more prevalent later-on. Blood metabolite profiles changed during the neonatal period, reflecting shifts in energy metabolism, immune response, and gut function as the calves transitioned from colostrum intake to solid feed. The temporal changes observed provide insights into the impacts of gut microbiota maturation on the metabolic health of calves, emphasizing the importance of gut-microbiome interactions during the early stages of life.

Animal welfare is another important aspect of farming that is known to significantly impact animal production. The impact of prolonged transportation on the welfare and health of goats is becoming a growing societal concern. The study by Batchu et al. used targeted metabolomics and demonstrated that extended transportation induces metabolic stress in goats, affecting key metabolic pathways related to amino acids metabolism, energy and stress response. However, habituation to livestock trailers appears to reduce the severity of these effects, suggesting that habituation may be a valuable strategy to improve the welfare and health of livestock during transportation. By identifying specific metabolic markers of stress, the findings offer practical insights into improving livestock management practices and ensuring animal welfare during transport.

Application of metabolomics has expanded in several livestock species, including pigs. For example, combining genomics and metabolomics, Dervishi et al. investigated the genetic and metabolic factors that influence the immune response. Analysis of genetic correlations between plasma metabolites and complete blood count (CBC) traits in young pigs revealed that plasma concentration of L-proline and L-glutamine were genetically positively correlated with hemoglobin and neutrophils concentration, respectively. In addition, metabolites such as dimethylglycine, betaine, and L-methionine were reported as candidate metabolites to improve growth rate of young healthy pigs. This research has practical implications for improving pig health and immunity through selective breeding strategies based on metabolic and blood traits. The study also opens up the potential for precision livestock farming through metabolic profiling to monitor and optimize immune function in pigs.

In this special edition, application of metabolomics expanded beyond livestock species. For example, in bats, echolocation is a complex biological process that involves the production and detection of sound waves, and the cochlea plays a central role in this auditory system. The study by Wang et al. used untargeted metabolomics to identify and compare the metabolites in the cochleae of the two bat species. By identifying specific metabolites related to several biological processes, including signaling pathways, nervous system, and metabolic process, the study advances the understanding of how molecular mechanisms support complex biological functions like echolocation. These findings could have broader implications for studying auditory physiology and evolution in other echolocating mammals and other species.

In this special edition, studies on silkworms and parasitic nematodes that used metabolomics were included. Wu et al. investigated the impact of different diets on cocoon yield in silkworms. Traditionally, silkworms are fed mulberry leaves, which are known to be their natural diet. This study compared the effect of feeding formula feed and mulberry leaves on cocoon production and quality. Distinct metabolic profiles between silkworms fed with mulberry leaves and formula feed were observed in regards to cysteine and methionine metabolism, arginine biosynthesis, and arginine and proline metabolism. This study provides insights into the potential mechanisms through which formula feed may enhance silk production and could provide directions for formula feed optimization in factory-raised silkworms. On the other hand, Polak et al. examined how the parasitic nematode Anisakis simplex adapts to different host environments during its larval stages. Using metabolomic analysis, the study compared the metabolic profiles of Anisakis simplex larvae at various developmental stages, focusing on how these changes help the parasite survive and thrive inside different hosts. Metabolic pathways related to amino acids, starch, and sucrose were mainly activated in the L3 stage, meanwhile the molecules responsible for successful migration within their host, such as pyridoxine and prostaglandins (E1, E2, F1a) were present in the L4 stage. This research enhances the understanding of Anisakis simplex’s adaptation mechanisms to different host environments, providing valuable insights into parasite-host interactions. The findings may inform future strategies for controlling parasitic infections in humans and animals by targeting specific metabolic pathways crucial for the parasite’s survival.

The research articles presented in this Research Topic contribute to a deeper understanding of metabolic pathways and how they influence physiological, health, and disease states in different animal species. In addition, it highlights the potential of metabolomics to advance our knowledge of animal physiology and improve animal welfare and health, fertility and production practices.

